# Systematic and other reviews: criteria and complexities

**DOI:** 10.1186/s40463-021-00527-9

**Published:** 2021-07-01

**Authors:** Robert T. Sataloff, Matthew L. Bush, Rakesh Chandra, Douglas Chepeha, Brian Rotenberg, Edward W. Fisher, David Goldenberg, Ehab Y. Hanna, Joseph E. Kerschner, Dennis H. Kraus, John H. Krouse, Daqing Li, Michael Link, Lawrence R. Lustig, Samuel H. Selesnick, Raj Sindwani, Richard J. Smith, James Tysome, Peter C. Weber, D. Bradley Welling

**Affiliations:** 1 Editor-in-Chief, Journal of Voice, Philadephia, USA; 2 Editor Emeritus, Ear, Nose and Throat Journal, Philadephia, USA; 3 Assistant Editor, Otology & Neurotology, Lexington, USA; 4 Editor-in-Chief, Ear, Ear, Nose and Throat Journal, Nashville, USA; 5 Editor-in-Chief, Journal of Otolaryngology – Head & Neck Surgery, Toronto, Canada; 6 Editor-in-Chief, Journal of Otolaryngology – Head & Neck Surgery, London, Canada; 7 Senior Editor, Journal of Laryngology and Otology, Birmingham, UK; 8 Editor-in-Chief, Operative Techniques in Otolaryngology – Head and Neck Surgery, Hershey, USA; 9 Editor-in-Chief, Head & Neck, Houston, USA; 10 Editor-in-Chief, International Journal of Pediatric Otorhinolaryngology, Milwaukee, USA; 11 Co-Editor-in-Chief, Journal of Neurological Surgery Part B: Skull Base, New York, USA; 12 Editor-in-Chief, Otolaryngology – Head and Neck Surgery, Philadelphia, USA; 13 Editor-in-Chief, OTO-Open, Philadelphia, USA; 14 Editor-in-Chief, Journal for Oto-Rhino-Laryngology, Head and Neck Surgery, Philadelphia, USA; 15 Editor-in-Chief, World Journal of Otorhinolaryngology – Head and Neck Surgery, Philadelphia, USA; 16 Co-Editor-in-Chief, Journal of Neurological Surgery Part B: Skull Base, Rochester, USA; 17 Editor-in-Chief, Otology & Neurotology, New York, USA; 18 Editor-in-Chief, The Laryngoscope, New York, USA; 19 Editor-in-Chief, American Journal of Rhinology & Allergy, Cleveland, USA; 20 Editor-in-Chief, Annals of Otology, Rhinology & Laryngology, Iowa City, USA; 21 Editor-in-Chief, Clinical Otolaryngology, Cambridge, UK; 22 Editor-in-Chief, American Journal of Otolaryngology, Boston, USA; 23 Editor-in-Chief, Laryngoscope Investigative Otolaryngology, Boston, USA

Review articles can be extremely valuable. They synthesize information for readers, often provide clarity and valuable insights into a topic; and good review articles tend to be cited frequently. Review articles do not require Institutional Review Board (IRB) approval if the data reviewed are public (including private and government databases) and if the articles reviewed have received IRB approval previously. However, some institutions require IRB review and exemption for review articles. So, authors should be familiar with their institution’s policy. In assessing and interpreting review articles, it is important to understand the article’s methodology, scholarly purpose and credibility. Many readers, and some journal reviewers, are not aware that there are different kinds of review articles with different definitions, criteria and academic impact [1]. In order to understand the importance and potential application of a review article, it is valuable for readers and reviewers to be able to classify review articles correctly.

## Systematic reviews

Authors often submit articles that include the term “systematic” in the title without realizing that that term requires strict adherence to specific criteria. A systematic review follows explicit methodology to answer a well-defined research question by searching the literature comprehensively, evaluating the quantity and quality of research evidence rigorously, and analyzing the evidence to synthesize an answer to the research question. The evidence gathered in systematic reviews can be qualitative or quantitative. However, if adequate and comparable quantitative data are available then a meta-analysis can be performed to assess the weighted and summarized effect size of the studies included. Depending on the research question and the data collected, systematic reviews may or may not include quantitative meta-analyses; however, meta-analyses should be performed in the setting of a systematic review to ensure that all of the appropriate data were accessed. The components of a systematic review can be found in an important article by Moher et al. published in 2009 that defined requirements for systematic reviews and meta-analyses [2].

In order to optimize reporting of meta-analyses, an international group developed the Quality of Reporting of Meta-Analyses (QUOROM) statement at a meeting in 1996 that led to publication of the QUOROM statement in 1999 [3]. Moher et al. revised that document and re-named the guidelines the Preferred Reporting Items for Systematic Reviews and Meta-Analyses (PRISMA). The PRISMA statement included both meta-analyses and systematic reviews, and the authors incorporated definitions established by the Cochrane Collaboration [4]. The PRISMA statement established the current standard for systematic reviews. To qualify as a systematic review, the methods section should acknowledge use of the PRISMA guidelines, and all PRISMA components should be incorporated strictly in all facets of the paper from the research question to the discussion. The PRISMA statement includes a checklist of 27 items that must be included when reporting a systematic review or meta-analysis [2]. A downloadable version of this checklist can be used by authors, reviewers, and journal editorial staff to ensure compliance with recommended components [5]. All 27 will not be listed in this brief editorial (although authors and reviewers are encouraged to consult the article by Moher et al. and familiarize themselves with all items), but a few will be highlighted.

The research question, as reflected in the title, should be a hypothesis-based specific research inquiry. The introduction must describe the rationale for the review and provide a specific goal or set of goals to be addressed. The type of systematic review, according to the Cochrane Collaboration, is based on the research question being asked and may assess diagnostic test accuracy, review prognostic studies evidence, evaluate intervention effect, scrutinize research methodology, or summarize qualitative evidence [6].

In the methods section, the participants, interventions, comparisons, outcomes and study design (PICOS) must be put forward. In addition to mentioning compliance with PRISMA, the methods section should state whether a review protocol exists and, if so, where it can be accessed (including a registration number). Systematic reviews are eligible for registration in the International Prospective Register of Systematic Reviews (PROSPERO) as established at the University of York (York, UK). When PROSPERO is used (it is available but not required for systematic reviews), registration should occur at the initial protocol stage of the review, and the final paper should direct to the information in the register. The methods section also must include specific study characteristics including databases used, years considered, languages of articles included, specific inclusion and exclusion criteria for studies; and rationale for each criterion must be included. Which individuals specifically performed searches should be noted. Electronic search strategy (with a full description of at least one electronic search strategy sufficient to allow replication of the search), process for article selection, data variables sought, assumptions and simplifications, methods for assessing bias risk of each individual study (such as selective reporting in individual studies) and utilization of this information in data synthesis, principal summary measures (risk ratio, hazard ratio, difference in means, etc.), methods of data management and combining study results, outcome level assessment, and other information should be reported.

The results section should include the number of studies identified, screened, evaluated for eligibility (including rationale for exclusion), and those included in the final synthesis. A PRISMA flow diagram should be included to provide this information succinctly [7]. The results also should include the study characteristics, study results, risk of bias within and across studies, and a qualitative or quantitative synthesis of the results of the included studies. This level of rigor in acquiring and evaluating the evidence of each individual study is one of the criteria that distinguishes systematic reviews from other categories. If the systematic review involves studies with paired samples and quantitative data, a summary of data should be provided for each intervention group along with effect estimates and confidence intervals for all outcomes of each study. If a meta-analysis is performed, then synthesized effect size should be reported with confidence intervals and measures of consistency (i.e. – data heterogeneity such as I^2^) for each meta-analysis, and assessment of bias risk across studies. A forest plot, which provides a graphical presentation of the meta-analysis results, should be included.

The discussion section should summarize the main findings commenting on the strength of evidence for each outcome, as well as relevance to healthcare providers, policymakers and other key stake-holders; limitations of the study and outcomes; and conclusions highlighting the interpretation of results in the context of other research, and implications for future research.

Without adhering to of all of these criteria and the others listed in the PRISMA statement and checklist, the review does not qualify to be classified as “systematic”.

## Meta-analyses

Meta-analyses, when feasible based on available and comparable quantitative data, supplement a systematic review evaluation, by adding a secondary statistical analysis of the pooled weighted outcomes of similar studies. This adds a level of objectivity in the synthesis of the review’s findings. Meta-analyses are appropriate when at least 2 individual studies contain paired samples (experimental group and control group) and provide quantitative outcome data and sample size. Studies that lack a control group may over-estimate the effect size of the experimental intervention or condition being studied and are not ideal for meta-analyses [8]. It also should be remembered that the conclusions of a meta-analysis are only as valid as the data on which the analysis is based. If the articles included are flawed, then the conclusions of the meta-analysis also may be flawed. Systematic reviews and meta-analyses are the most rigorous categories of review.

## Other types of reviews

### Mixed methods reviews

Systematic reviews typically contain a single type of data, either qualitative or quantitative; however, mixed methods reviews bring together a combination of data types or study types. This approach may be utilized when quantitative data, in the setting of an intervention study, only provide a narrow perspective of the efficacy or effectiveness of the intervention. The addition of qualitative data or qualitative studies may provide a more complete picture of the knowledge, attitudes, and behaviors of clinicians, patients or researchers regarding that intervention. This type of review could involve collecting either the quantitative or the qualitative data using systematic review methodology, but often the qualitative data are gathered using a convenience sampling. Many qualitative studies provide useful insights into clinical management and/or implementation of research interventions; and incorporating them into a mixed methods review may provide valuable perspective on a wide range of literature. Mixed methods reviews are not necessarily systematic in nature; however, authors conducting mixed methods reviews should follow systematic review methodology, when possible.

### Literature and narrative reviews

Literature reviews include peer-reviewed original research, systematic reviews, and meta-analyses, but also may include conference abstracts, books, graduate degree theses, and other non-peer reviewed publications. The methods used to identify and evaluate studies should be specified, but they are less rigorous and comprehensive than those required for systematic reviews. Literature reviews can evaluate a broad topic but do not specifically articulate a specific question, nor do they synthesize the results of included studies rigorously. Like mixed method reviews, they provide an overview of published information on the topic, although they may be less comprehensive than integrative reviews; and, unlike systematic reviews, they do not need to support evidence-based clinical or research practices, or highlight high-quality evidence for the reader. Narrative reviews are similar to literature reviews and evaluate the same scope of literature. The terms sometimes are used interchangeably, and author bias in article selection and data interpretation is a potential concern in literature and narrative reviews.

### Umbrella reviews

An umbrella review integrates previously published, high-quality reviews such as systematic reviews and meta-analyses. Its purpose is to synthesize information in previously published systematic reviews and meta-analyses into one convenient paper.

### Rapid review

A rapid review uses systematic review methodology to evaluate existing research. It provides a quick synthesis of evidence and is used most commonly to assist in emergent decision-making such as that required to determine whether COVID-19 vaccines should receive emergent approval.

### Scoping, mapping, and systematized reviews

If literature has not been reviewed comprehensively in a specific subject that is varied and complex, a mapping review (also called scoping review) may be useful to organize initial understanding of the topic and its available literature. While mapping reviews may be helpful in crystallizing research findings and may be published, they are particularly useful in helping to determine whether a topic is amenable to systematic review, and to help organize and direct the approach of the systematic review or other reviews of the subject. Systematized reviews are used most commonly by students. The systematized review provides initial assessment of a topic that is potentially appropriate for a systematic review, but a systematized review does not meet the rigorous criteria of a systematic review and has substantially more limited value. Additional types of reviews exist including critical review, state-of-the-art review, and others.

## Conclusion

Reviews can be invaluable; but they also can be misleading. Systematic reviews and meta-analyses provide readers with the greatest confidence that rigorous efforts have attempted to eliminate bias and ensure validity, but even they have limitations based upon the strengths and weaknesses of the literature that they have assessed (and the skill and objectivity with which the authors have executed the review). Risks of bias, incomplete information and misinformation increase as the rigor of review methodology decreases. While review articles may summarize research related to a topic for readers, non-systematic reviews lack the rigor to answer adequately hypothesis-driven research questions that can influence evidence-based practice. Journal authors, reviewers, editorial staff, and should be cognizant of the strengths and weaknesses of review methodology and should consider them carefully as they assess the value of published review articles, particularly as they determine whether the information presented should alter their patient care.
